# Human APOBEC3A Isoforms Translocate to the Nucleus and Induce DNA Double Strand Breaks Leading to Cell Stress and Death

**DOI:** 10.1371/journal.pone.0073641

**Published:** 2013-08-20

**Authors:** Bianka Mussil, Rodolphe Suspène, Marie-Ming Aynaud, Anne Gauvrit, Jean-Pierre Vartanian, Simon Wain-Hobson

**Affiliations:** Molecular Retrovirology Unit, Institut Pasteur, Paris, France; Institut Pasteur Korea, Korea, Republic Of

## Abstract

Human APOBEC3 enzymes deaminate single stranded DNA. At least five can deaminate mitochondrial DNA in the cytoplasm, while three can deaminate viral DNA in the nucleus. However, only one, APOBEC3A, can hypermutate genomic DNA. We analysed the distribution and function of the two APOBEC3A isoforms p1 and p2 in transfected cell lines. Both can translocate to the nucleus and hypermutate *CMYC* DNA and induce DNA double strand breaks as visualized by the detection of ©H2AX or Chk2. APOBEC3A induced G1 phase cell cycle arrest and triggered several members of the intrinsic apoptosis pathway. Activation of purified human CD4+ T lymphocytes with PHA, IL2 and interferon α resulted in C->T hypermutation of genomic DNA and double stranded breaks suggesting a role for APOBEC3A in pro-inflammatory conditions. As chronic inflammation underlies many diseases including numerous cancers, it is possible that APOBEC3A induction may generate many of the lesions typical of a cancer genome.

## Introduction

The human apolipoprotein-B-mRNA-editing catalytic polypeptide-like 3 (*APOBEC3*) locus consists of seven genes (*A3A-A3H*) that encode single-stranded DNA cytidine deaminases [1]. They are part of a larger group of polynucleotide cytidine deaminases (PCDs) that include the mRNA editor APOBEC1 and activation-induced deaminase (AID). AID is an ssDNA mutator [2] essential for class-switch recombination and somatic hypermutation of rearranged immunoglobulin genes in B cells [3,4]. However, off-target AID activity can cause non-specific deamination inducing double strand breaks (DSBs) throughout the cellular genome [5,6] and cancers in transgenic mice [7–12]. Such double strand breaks (DSBs) give rise to the characteristic translocations found in B-cell lymphomas [13].

The *APOBEC3* locus is unique to placental mammals and arose from duplication and subsequent expansion of the *AID* locus [14]. Several APOBEC3 enzymes act as restriction factors for retroviruses [15–21]. They target retroviral cDNA intermediates and deaminate cytidine to uridine, effectively leading to lethal mutagenesis. This was such a problem for precursor lentiviruses that they evolved the vif gene to circumvent APOBEC3G (A3G) and APOBEC3F (A3F) [22–27].

Human hepatitis B virus DNA is vulnerable to editing by several APOBEC3 enzymes leading to impaired HBV replication, although A3C and A3G are probably the important PCDs *in vivo* [28,29]. DNA viral genomes too can undergo editing, for example human papillomavirus (HPV) DNA was found to be vulnerable to A3 editing *in vivo* and by A3A, A3C and A3H *ex vivo* [30] while herpes simplex virus type 1 was particularly susceptible to A3C [31]. The induction of numerous *A3* genes by interferon-α in many cell types fitted with an antiviral role [32–39].

Despite their antiviral roles and the AID paradigm, it was always possible that non-infectious phenomena could be linked to some APOBEC3 PCDs. It was reported that A3A, A3C and A3H enzymes could extensively edit transfected plasmid DNA in human cells [30,36], while five A3 enzymes were found to edit single-stranded mitochondrial DNA (mtDNA) in the cytoplasm. APOBEC3A (A3A) can access and edit nuclear DNA (nuDNA), suggesting a role for APOBEC3 enzymes in DNA catabolism and perhaps cancer [40]. Recently it was suggested that APOBEC3B could be a source of C->T mutations in breast cancer genomes [41–43]. As individuals with the homozygous deletion for *APOBEC3B* have a higher odds ratio of developing breast and liver cancer, an extra layer of complexity surrounding the gene needs to be fathomed [44,45].

A3A can lead to DNA damage and cell cycle arrest in U2OS cells [46], while A3A could induce mutations in ssDNA during *in vitro* transcription, the non-transcribed strand being transiently single-stranded [47]. Deamination of genomic DNA results in DNA enriched with uracil, which activates base excision repair (BER). Uracil DNA-glycosylase (UNG) excises uracil and abasic endonucleases cleave the DNA strand leading to repair or degradation. However, DSBs can be generated during repair of two mutations in a cluster [48] where two such breaks occur in close proximity on opposite strands. Immediately following DSB formation, PI3K-like kinases, a family including ataxia telangiectasia mutated (ATM), ataxia telangiectasia and Rad3-related (ATR) and DNA-dependent protein kinase (DNA-PK), are activated and phosphorylate H2AX at serine 139 leading to the formation of γH2AX [49–54]. Many other DNA repair and cell cycle checkpoint proteins, such as Chk1 and Chk2, are also activated enhancing the DNA damage signal [55]. DSBs are considered to be the most serious type of DNA damage and a few of these lesions are sufficient to induce gene mutations, chromosomal aberrations and cell transformation [56]. Unrepaired DSBs invariably induce apoptosis [57].

Here we quantified the nuclear translocation for the two A3A isoforms and investigated their potential to induce mutations and DSBs in nuDNA. It transpires that nuclear DNA is vulnerable to editing by both A3A isoforms leading to γH2AX positive DSBs, Chk2 phosphorylation and G1 phase cell cycle arrest accompanied with cell death. Hypermutation and DSBs in stimulated CD4+ T lymphocytes further indicate a role for A3A *in vivo* under inflammatory conditions.

## Materials and Methods

### A3A isoforms p1 and p2

The cDNAs encoding the two A3A isoforms were those corresponding to the sequence Genbank Accession Number NM_145699. Primers were designed to equip both A3A isoforms with adequate and strong Kozak motifs respectively. For one construction the SV40 TAg nuclear localization signal (NLS, residues PPKKKRKV) was added to the C-terminus. Full-length cDNAs were subcloned in the pcDNA3.1D/V5-His-TOPO expression vector (Invitrogen). All constructs were transformed and amplified in *E. coli* DH5α strain. Catalytically inactive A3A mutants were made by engineering C101S or C106S substitutions into active site residues, following manufacturer’s recommendation (GeneArt Site-Directed Mutagenesis System, Invitrogen).

### Cell Culture and Transfection

HeLa cells (ATCC CCL2) were grown in Dulbecco’s modified Eagle’s medium (DMEM) (Gibco Invitrogen), supplemented with 10% heat-inactivated fetal calf-serum (PAA), 50 U/ml penicillin and 50 µg/ml streptomycin (Gibco Invitrogen). The Japanese quail muscle fibroblast cell line QT6 (ATCC CRL 1708) was maintained in HAM’s F40 medium (Eurobio), supplemented with 50 U/ml penicillin, and 50 µg/ml streptomycin, 10% heat-inactivated fetal calf serum, 2 mM L-glutamine (Sigma), 5% tryptose phosphate (Eurobio) and 1% chicken serum (PAA). Cells were grown as monolayers in 75 cm^2^ cell culture flasks at 37°C in a humidified atmosphere containing 5% CO_2_.

For transfection, either 5x10^5^ HeLa cells or 7x10^5^ QT6 cells were seeded in six-well tissue culture plates and incubated for 24 h. Transfections were performed with equal amounts of individual expression plasmids using jetPRIME (Polyplus transfection) or FugeneHD (Roche). Cells treated for 16 h with 100 µM etoposide (Sigma Aldrich) or 100 µM actinomycin D (Sigma Aldrich) served as positive controls. At 24 h or 48 h after transfection, cells were trypsinized (Gibco Invitrogen), floating and adherent cells were collected, washed with cold PBS (Gibco Invitrogen) and analysed for DSBs and apoptosis.

### Isolation and activation of CD4+ T lymphocytes

Blood was obtained from anonymous healthy donors (Authorisation IP: HS2004-3165 and HS2012-24917) and approved by the Comité Consultatif National d’Ethique (CCNE) de la Direction Générale et d’instances éthique et déontologique de l’Institut Pasteur. Federalwide Assurance (FWA) for the Protection of Human Subjects is FWA00003327 (N° IRB : 00006966). The anonymous healthy donors provided their written informed consent to participate in this study. Peripheral blood mononuclear cells (PBMC) were isolated by Ficoll gradient (Eurobio). Isolation of CD4+ T lymphocytes was performed by incubation with antibody-coated magnetic beads (Miltenyl Biotec). Purity of CD4+ T lymphocytes was above 90% as checked by flow cytometry (FACSCalibur, Becton Dickinson). CD4+ T lymphocytes were stimulated with 10 µg ml^-1^ PHA (Sigma), 100 U ml^-1^ IL2 (Sigma) and 1000 U ml^-1^ IFN-〈 (PBL biomedical laboratories) for 48 h.

### PCR and 3DPCR

All DNAs were extracted using the MasterPure Complete DNA and RNA purification kit (EPICENTRE Biotechnologies). Amplifications were performed in a first-round standard PCR followed by nested 3DPCR with 2.5 U Taq (Bioline) DNA polymerase per reaction. PCR products were cloned using the TOPO TA Cloning kit (Invitrogen) while sequencing was outsourced to GATC. PCR conditions and primers have been described [40]. For the detection of hypermutation by 3DPCR [58], primary cells were infected with lentivirus rV2.EF1.UGI, which encodes a codon optimized UNG inhibitor (UGI) under the control of the constitutive human EF1 promoter generated by Vectalys (Toulouse, France). Stock virus was pseudotyped with the VSV G protein. Purified human CD4+ T lymphocytes were transduced by polybrene (Santa Cruz Biotechnology) at the MOI of five according to the manufacturer’s instruction.

### Flow cytometry of DNA damage response (DDR)

Twenty-four and 48 h post transfection floating and adherent cells were washed with PBS, fixed in 2% ice-cold paraformaldehyde (Electron Microscopy Sciences) for 15 min and permeabilized in 90% ice-cold methanol (Sigma) for 30 min. After two washes with PBS, cells were incubated for 1 h with 1:200 diluted mouse anti-V5 antibody (Invitrogen). DNA double strand breaks were analysed by staining for 1 h with 1:50 diluted Alexa Fluor 488-conjugated rabbit monoclonal anti-©H2AX (20E3) antibody (Cell Signaling). Phosphorylated Chk2 was detected by using 1:50 diluted rabbit monoclonal anti-Chk2-P (C13C1) antibody (Cell Signaling) for 1 h. Detection of cleaved PARP was performed by incubation with 1:50 diluted Alexa Fluor 488-conjugated rabbit monoclonal anti-cleaved PARP (D64E10) antibody (Cell Signaling). Following secondary antibodies were: 1:500 diluted Alexa Fluor 633 F(ab’)_2_ fragment of goat anti-mouse IgG (H+L) (Invitrogen), 1:100 diluted FITC goat anti-mouse IgG (Sigma) or 1:100 diluted FITC goat anti-rabbit IgG (Sigma) for 45 min. All incubation steps were performed on ice. Cells were analysed on FACSCalibur (BD Biosciences) using CellQuest Pro (BD Biosciences, version 5.2) or FlowJo software (Tree Star, Inc., version 8.7.1). For each sample 10,000 cells were counted.

### ImageStream analysis

At 24 h post transfection HeLa cells were fixed, permeabilized and stained as described for flow cytometry. After staining of nuclei with DAPI, cells were analyzed on an ImageStream multispectral flow cytometer and images were analyzed using IDEAS image-analysis software (Amnis Corporation). Ten thousand events were collected in each sample and single stained controls were used to compensate fluorescence between channel images on a pixel-by-pixel basis. The instrument combines the features of classic fluorescence microscopy and flow cytometry so allowing multiparametric analyses [59]. The machine enabled gating around single cells, allowing detailed morphological analysis based on acquired cellular images. Nuclear translocation of A3A was determined by using the similarity feature in the IDEAS software. The similarity score (a monotonic function of Pearson’s correlation coefficient between the pixel values of two image pairs) provides a measure of the degree of nuclear localization of A3A by computing the pixel intensity correlation between the nuclear image (DAPI) and the translocated probe (APOBEC-V5 anti-V5 Alexa). Cells with low similarity scores exhibit no correlation of the images (hence a cytoplasmic distribution), whereas cells with high scores exhibit a positive correlation of the images (hence a nuclear distribution). Quantification of DSBs was performed using the similarity score between ©H2AX Alexa Fluor 488 spots and DAPI images.

### Cell cycle analysis

HeLa cells were transfected for 24 h. RNA was removed with RNase A and DNA was stained with propidium iodide (PI) according to manufacturer’s instructions of Cell Cycle Kit (Genscript). Cells were analysed with FACSCalibur using Cell Quest Pro or FlowJo software. For each sample 10,000 events were collected. Cellular aggregates and debris were excluded from analysis by proper gating. Data were fit to define the G1, S, and G2/M phases by using the Dean-Jett-Fox mathematical model of the FlowJo software.

### Mitochondrial cytochrome c release

At 24 h post transfection, HeLa cells were trypsinized and investigated for cytochrome c release by using the FlowCellect cytochrome c kit from Millipore according to manufacturer’s instructions. Cells were analysed with FACSCalibur using Cell Quest Pro or FlowJo software. For each sample 10,000 cells were counted.

### Western blotting

Twenty-four hours post transfection, HeLa cells were homogenized in ice-cold RIPA buffer containing protease inhibitors, PMSF and sodium orthovanadate (cell lysis buffer kit from Santa Cruz Biotechnology). The homogenate was clarified by centrifugation and the total protein amount of supernatant was determined by using the Bradford method (Bio-Rad). Equal amounts of proteins (40 to 60 µg) were mixed with LDS Sample Buffer (Invitrogen) and Sample Reducing Agent (Invitrogen), heated for 10 min at 95°C and were subjected to 4-12% Bis-Tris Gel (Invitrogen) at 125 V in MES SDS Running Buffer (Invitrogen). The bands were electrotransferred to nitrocellulose membranes (Amersham Biosciences) in Transfer Buffer (Invitrogen) for 1 h at 100 mA.

Membranes were blocked for 1 h with 5% non-fat dry milk in PBS containing 0.1% Tween-20 (Merck) (PBST). Membranes were incubated overnight at 4°C with primary antibodies. These were: 1:500 diluted rabbit monoclonal anti-cleaved caspase-3 (Asp175) (5A1E) antibody (Cell Signalling) and 1:1000 diluted rabbit monoclonal anti-tubulin antibody (Cell Signalling). All antibodies were diluted in 5% non-fat dry milk with PBST. After incubation with the respective 1:2000 diluted goat horseradish peroxidase-conjugated secondary antibody (GE Healthcare), membranes were subjected to detection by ECL detection Supersignal West Pico Chemiluminescent Substrate (Thermo Scientific). For the detection of®-actin 1:25000 diluted anti-®-actin antibody cross-linked to horseradish peroxidase (Sigma) was used. For the detection of V5 1:5000 diluted anti-V5 antibody cross-linked to horseradish peroxidase (Invitrogen) was applied.

### FACS analysis of apoptosis

Annexin V possesses high affinity for the phospholipid phosphatidylserine (PS) thus identifying cells undergoing apoptosis [60]. At 24 h after transfection, HeLa cells were resuspended in binding buffer (BD Pharmingen) and stained with FITC-labelled Annexin V antibody (1 µg/ml) (BD Pharmingen). Cells were counterstained 5 µg/ml PI (BD Pharmingen) to distinguish between early apoptotic and late apoptotic or necrotic events. Cells were analysed with FACSCalibur using CellQuest Pro or FlowJo software. For each sample 10,000 events were collected.

### Statistical analyses

The statistical analyses were calculated with GraphPad Prism version 5 (GraphPad software). For comparison between two groups the nonparametric one tailed Mann–Whitney’s U test was used and for interpretation between more than two groups the Kruskal-Wallis test was used. The confidence intervals were set at 95%. For correlation the nonparametric two tailed Spearman test was performed. Significance level was always set at p-values less than 0.05.

## Results

### A3A isoforms and nuclear translocation

**Figure 1:**
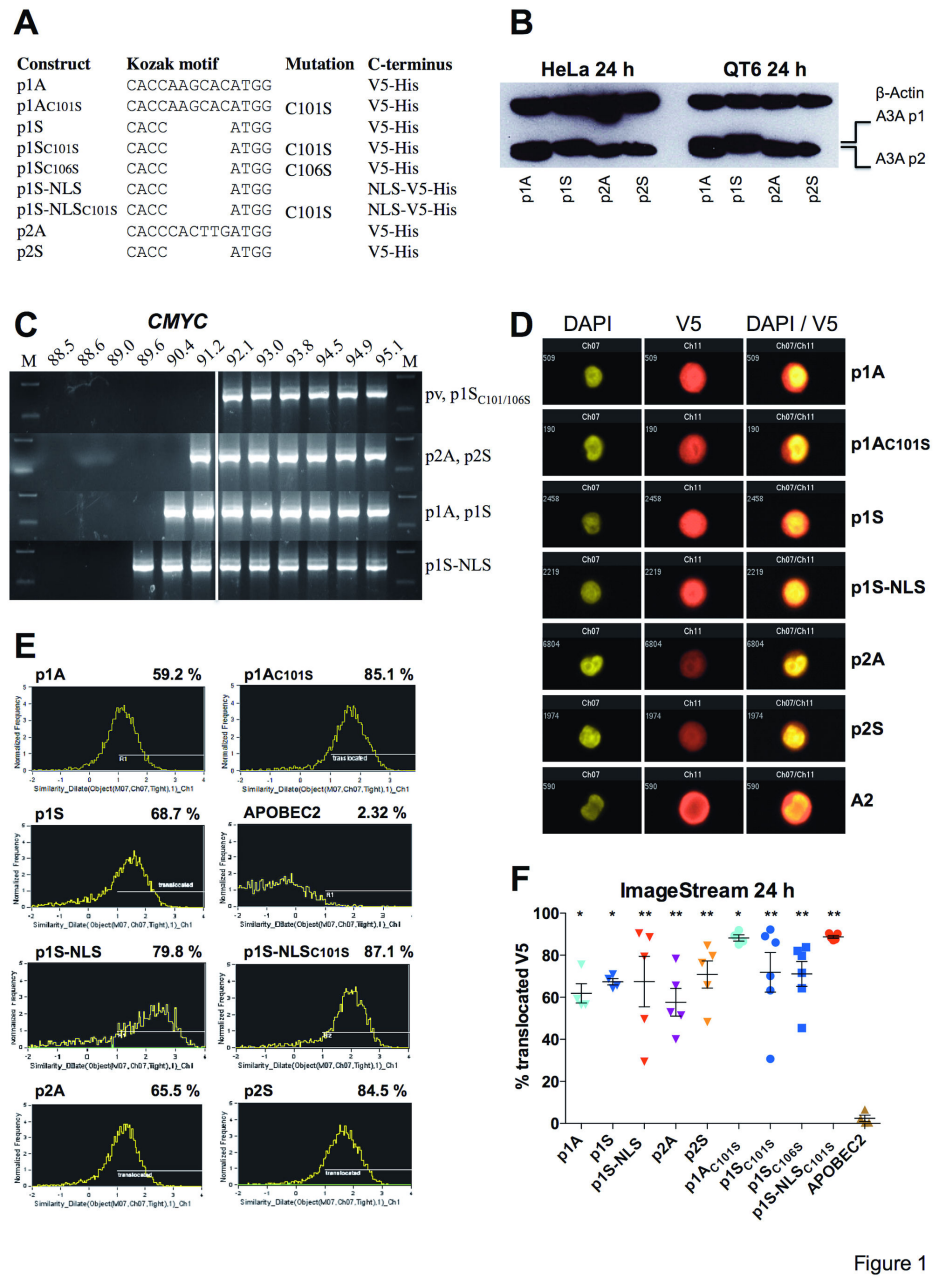
A3A isoforms and nuclear translocation. (A) A3A constructs with natural adequate (A) and strong (S) Kozak contexts. The human A3A sequence (NM_145699) allows translation initiation at codons 1 and 13 giving rise to two functional isoforms, p1 and p2. Both A3A isoforms (p1 and p2) were presented with adequate (A) and strong (S) Kozak motifs respectively. For p1S-NLS and p1S-NLS_C101S_, the SV40 TAg nuclear localization signal (NLS, residues PPKKKRKV) was added to the C-terminus. p1S_C101S,_ p1S_C106S_ and p1A_C101S_ correspond to the catalytic mutants. A nuclear localization signal (NLS) was added at the C-terminus of p1S. (B) Western blot of the principal A3A constructs in HeLa and quail QT6 cells at 24 h post transfection. (C) All A3A constructs hyperedited human *CMYC* DNA. The 3DPCR gradient was 95°C to 88°C. The white line indicates the divide between unedited DNA (92.1°C and greater) and edited DNA (91°C and lower); pv, empty plasmid vector control. (D) ImageStream images of individual HeLa cell nuclei stained for DAPI and A3A-V5 constructs as well as merged images. (E) Population-based readouts for ImageStream data and frequency for nuclear translocated A3A-V5 tagged constructs. (F) Proportion of V5-tagged APOBEC constructs translocated to the nucleus. The amount of translocated APOBEC to the nucleus was calculated by using the similarity score feature of the IDEAS software between the nuclear image (DAPI) and the translocated probe (APOBEC-V5). Dots are representative for independent experiments. Mean and SEM are shown for between 4–6 independent experiments. Group differences to APOBEC2 were calculated using the Mann-Whitney test (*p< 0.05; **p< 0.01).

The human A3A sequence (NM_145699) allows translation initiation at codons 1 and 13 giving rise to two functional isoforms, p1 and p2 [61], the Kozak context of both initiator methionines being described as adequate (A). We generated a variety of constructs using both the natural Kozak sequences as well as those with strong (S) Kozak contexts (Figure 1A). A nuclear localization signal (NLS) was added at the C-terminus of p1S to enhance nuclear accumulation. All sequences were cloned in TOPO3.1 V5-tagged expression vector. Western blot analysis showed as expected that the natural construct p1A gave rise to the two isoforms p1 and p2, while p1S generated only the p1 isoform in both HeLa and the quail cell line QT6 (Figure 1B). Despite this there was no important difference in the steady state amount of protein produced at 24 hours. Similarly the p2A and p2S constructs produced comparable amounts of protein (Figure 1B). This shows that comparison of p1S and p2S should allow differentiation, if any, between the two isoforms.

All the constructs were able to edit human *CMYC* DNA (Figure 1C) as expected from the previously reported A3A p1S construct [40]. P1A and p1S appeared slightly more active than p2A and p2S while the different Kozak contexts impacted little nuDNA editing. P1S-NLS was the most active. The corresponding A3AC101S or C106S catalytic mutants were inactive. The 3DPCR technique is not a fully quantitative technique and so small differences are not informative.

HeLa cells were transfected with A3A-V5-tagged plasmids and analysed by ImageStream technology, which combines the quantitative advantages of common flow cytometry together with qualitative imagery. Images for individual cells can be visualized, for example Figure 1D shows individual DAPI positive nuclei expressing A3A-V5. The raw data for an ensemble of cells are shown in Figure 1E and the average numbers of A3A-V5 positive nuclei for 4-6 independent experiments are shown in Figure 1F. All A3A constructs translocated to the nucleus although there was considerable variance occasionally. APOBEC2 was used as negative control and was predominantly localized to the cytoplasm (Figure 1D–F).

### A3A Expression Leads to DNA DSBs

**Figure 2:**
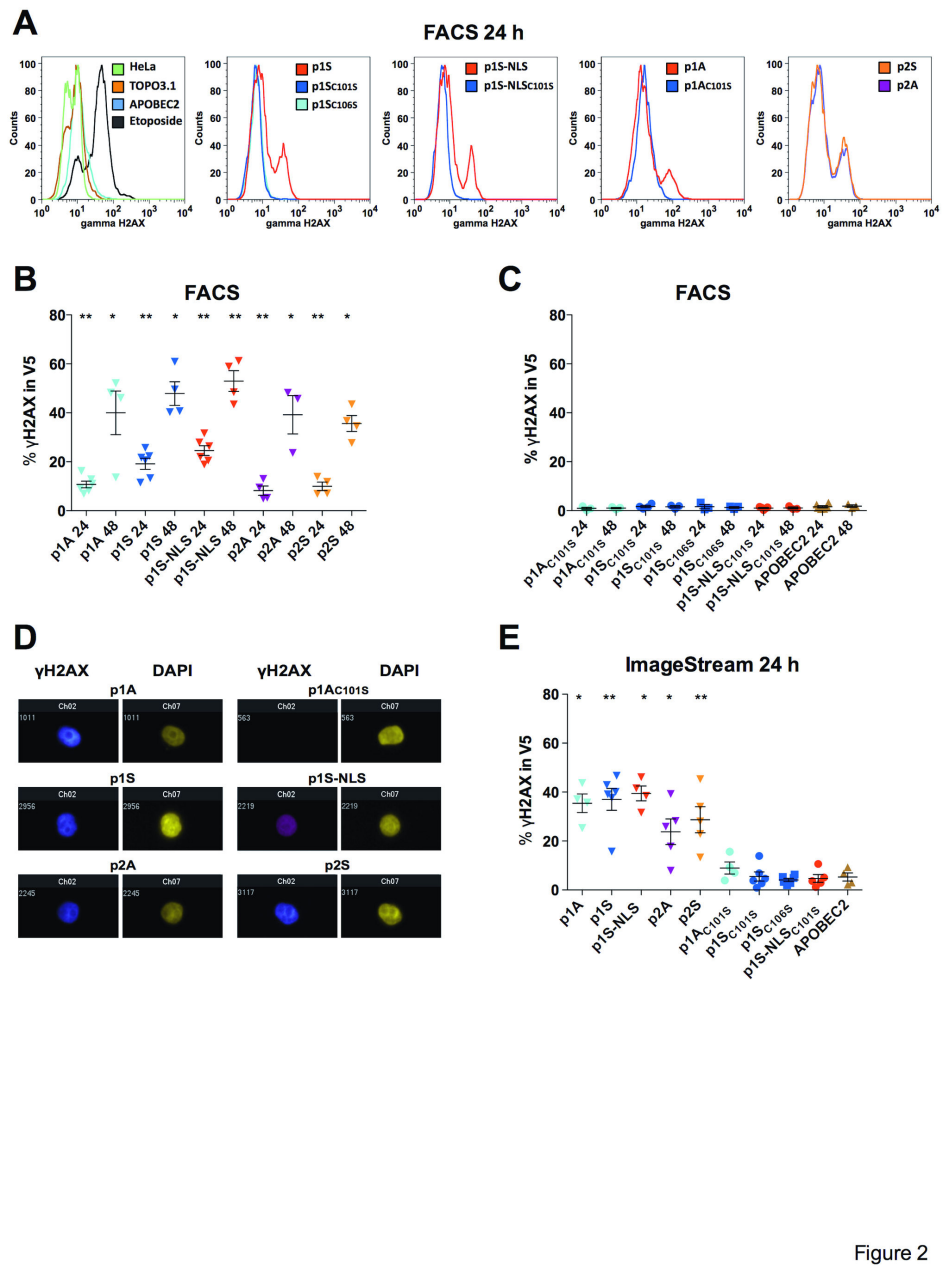
A3A expression leads to ©H2AX positive DSBs in HeLa cells. (A) Flow cytometry analysis of A3A induced DSBs at 24 h post transfection. (B) (C) Plots of γH2AX gated on V5 expressing cells for 4-6 independent experiments 24 and 48 h post transfection. The means and SEMs are shown. Group differences to APOBEC2 at 24 and 48 h were calculated using the Mann-Whitney test (*p< 0.05; **p< 0.01). (D) Individual nuclei showing γH2AX positive DSBs and DAPI 24 h post transfection. (E) Percentage of ©H2AX among A3A-V5 positive cells at 24 h post-transfection for 4-6 independent experiments. Mean and SEM are shown for between 4–6 independent experiments. Group differences to APOBEC2 were calculated using the Mann-Whitney test (*p< 0.05; **p< 0.01).

To quantitate A3A activity in the nucleus, we assessed genomic DNA damage by analysis of histone variant H2AX phosphorylation at serine 139 (γH2AX), a well known marker for DSBs and DNA damage response [62]. HeLa cells were transfected with A3A constructs while empty vector TOPO3.1 and APOBEC2 plasmid were used as negative controls. DSB induction with etoposide served as positive control [63] (Figure 2A and 2B). As can be seen from Figure 2A we found increased levels of DSBs in cells transfected with the p1S, p1A, p2S and p2A constructs, while the inactive cysteine mutants showed levels comparable to those from negative controls (untransfected cells and cells transfected with TOPO3.1 or APOBEC2). At 24 h the levels were highest for p1S and p1S-NLS (Figure 2B). After 48 h γH2AX levels were ~40-50% for all functional constructs (Figure 2B). No DSBs were observed in cells transfected with catalytically inactive A3A mutants or APOBEC2 (Figure 2C). The same was true for non-transfected cells or those transfected with the TOPO3.1 vector (Figures 2A and 7B).

Figure 2D shows selected images from individual transfected cells stained with DAPI showing the DSBs in the nucleus coincident with A3A nuclear translocation, while Figure 2E shows mean results presented as percentage of γH2AX in V5 expressing cells from 4–5 independent transfections. In accordance with flow cytometry data, increased levels of DSBs were found with all functional constructs. In contrast to the flow cytometry data, DSBs levels detected at 24 h were higher (approximately 30-40% γH2AX), probably due to different sensitivity of both systems.

**Figure 3:**
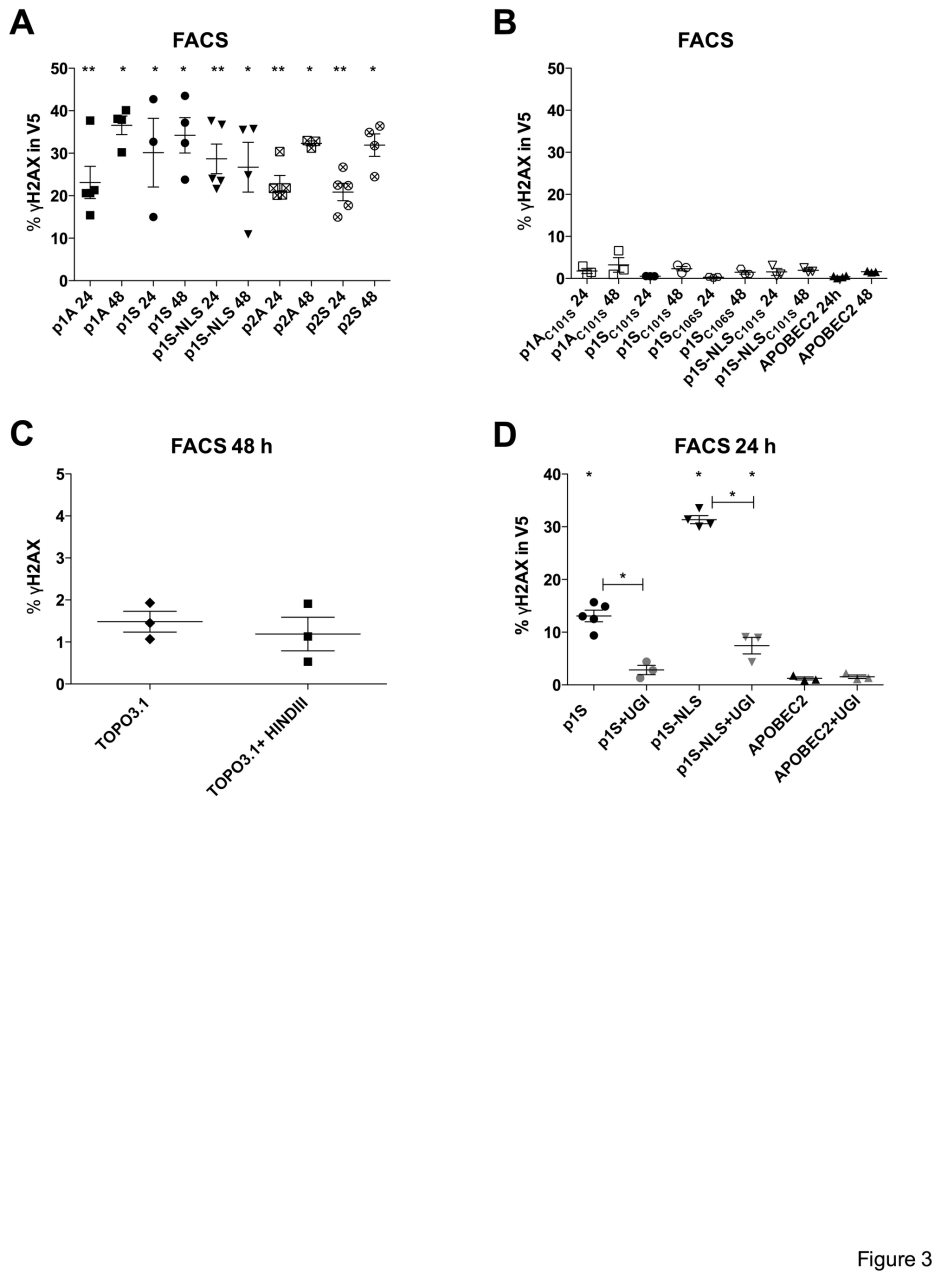
A3A expression leads to DSBs and requires UNG. (A) (B) A3A induces DSBs in the quail QT6 cell line at 24 and 48 h post-transfection respectively. Mean and SEM are shown for 4-5 independent experiments. Group comparisons to APOBEC2 at 24 and 48 h were calculated using the Mann-Whitney test (*p < 0.05; **p < 0.01). (C) DSBs originate from *de novo* genomic DNA damage. HeLa cells transfected with TOPO3.1 and HindIII cleaved TOPO3.1, which cleaves the vector just once were fixed. Mean and SEM are shown at 48 h post-transfection. (D) A3A induced DSBs require UNG cleavage of uracil. HeLa cells were transfected with APOBEC2, p1S and p1S-NLS alone and in the absence or presence of the UNG inhibitor (UGI) expressing plasmid. Mean and SEM are shown for 4-5 independent transfections at 24 h post-transfection. Group comparisons and differences to APOBEC2 were calculated using the Mann-Whitney test (*p < 0.05).

To exclude cell type specific effects, we analysed induction of DSBs in quail (QT6) cells. The avian lineage does not encode any A3 ortholog and does not show any cytidine deamination background [64,65]. Increased levels of γH2AX in V5 expressing cells were seen with A3A p1S, p1S-NLS, p1A, p2S and p2A 24 h post transfection (~25-30%, Figure 3A), which were slightly increased (25-35%) at 48 h (Figure 3A). Again, no DSBs were observed in cells transfected with catalytic inactive mutants, APOBEC2 (Figure 3B) as well as TOPO3.1 vector and non-transfected cells. To assess whether the observed DSBs are derived from genomic DNA or simply are resulting from sheared plasmid DNA or DSBs generated by A3A in plasmid DNA as opposed to nuDNA, we cleaved TOPO3.1 DNA with HindIII, which cleaves the plasmid just once. No induction of γH2AX was seen with cleaved and non-cleaved vector DNA (Figure 3C) in transfected HeLa cells, indicating the observed DSBs originate from *de novo* genomic DNA damage.

### A3A induced DNA DSBs require UNG

We have previously shown that A3A editing of nuDNA is rapidly followed by base excision repair enzymes initiated by uracil-DNA glycosylase (UNG) [40]. As this results in abasic sites, which can be subsequently cleaved by apurinic/apyrimidinic endonuclease, inhibition of UNG should reduce DSB formation. We transfected HeLa cells with p1S and p1S-NLS alone and in the presence of an UNG inhibitor (UGI) expressing plasmid [66]. In the presence of UGI a decrease in A3A-induced DSBs from 13% to 3% was noted for p1S and from 31% to 7% for p1S-NLS transfected cells (Figure 3D). The expression of UGI had no effect among cells transfected with APOBEC2 (Figure 3D) indicating that UNG plays an important role in the formation of DSBs after DNA editing.

### Induction of DNA DSBs and A3A editing in activated primary human CD4+ T lymphocytes

**Figure 4:**
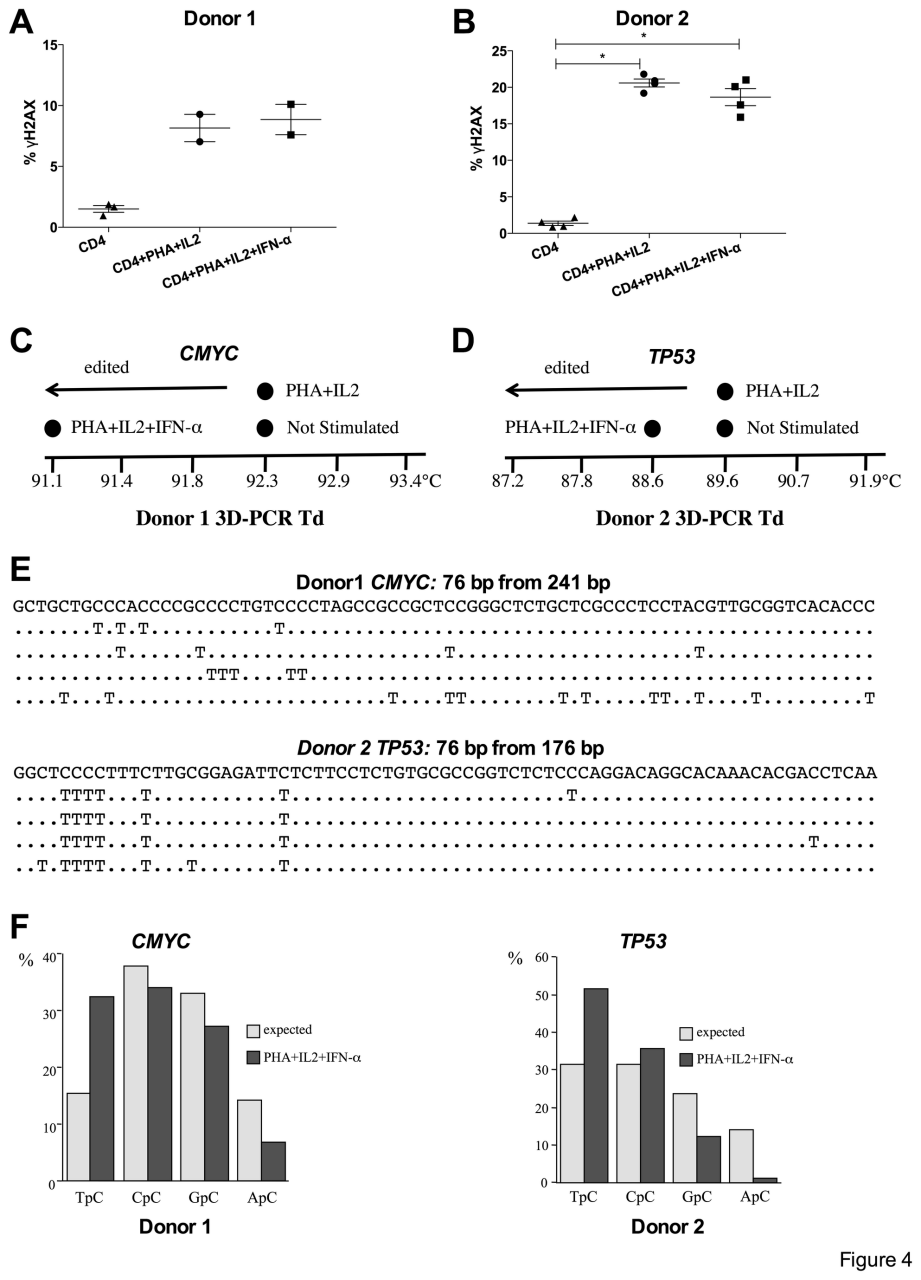
Induction of DSBs and A3A editing in activated primary human CD4+ T lymphocytes. (A) (B) ©H2AX positive DSBs in activated CD4^+^ T lymphocytes. Mean and SEM are shown. Group comparisons were calculated using the Mann-Whitney test (*p < 0.05). (C) (D) CD4+ T lymphocytes were transduced by recombinant lentivirus encoding the UNG inhibitor UGI (rV2.EF1.UGI). Recovery of hyperedited *CMYC* DNA by 3DPCR from donor 1 (C) and *TP53* DNA from donor 2 as shown by the denaturation temperature (Td) of the 3DPCR products (D). Only for the PHA+IL2+IFN-α treatment APOBEC3 edited DNA was recovered. The difference in minimal denaturation temperatures is due to the different base composition of the *CMYC* and *TP53* fragments. (E) A selection of hyperedited *CMYC* (Donor 1) or *TP53* (Donor 2) sequences respectively are shown compared to the unedited sequence. Only differences are shown. For space reasons only a fraction of the sequences are shown. (F) 5’ dinucleotide context associated with editing along with expected values assuming no editing bias. The clear preference for TpC is a diagnostic trait of A3A editing of nuDNA.

Transfected established tumour cell lines are hardly typical. To assess the potential of DNA damage in primary cells, we isolated CD4+ T lymphocytes from PBMC of two healthy donors and treated them with PHA, IL2 ± IFN-α, the latter being a known inducer of *A3A* expression [34,35,39,61,67,68]. Compared to untreated CD4+ T lymphoyctes, the levels of DSBs following PHA+IL2 and PHA+IL2+IFN-α stimulation were significantly increased, although levels appeared to be donor dependent (Figure 4A and B). As UNG activity is very efficient, detection of nuDNA editing by A3A requires UNG inhibition [40]. Accordingly CD4+ T lymphocytes were transduced by a recombinant lentivirus encoding a codon optimized UGI gene. Now, 3DPCR was able to recover *CMYC* and *TP53* DNA at restrictive temperatures following stimulation with PHA+IL2+IFN-〈 (Figure 4C and D). Sequence analysis showed large numbers of C->T induced mutations, a selection being shown in Figure 4E. Importantly the strong preference for editing associated with the TpC dinucleotide is diagnostic of A3A involvement (Figure 4F) [40]. The above data indicate that DSBs induction in primary CD4+ T lymphocytes emanated from A3A expression and suggests a role of A3A enzymes during immune responses.

### A3A expression induces DNA damage response and cell cycle arrest

**Figure 5:**
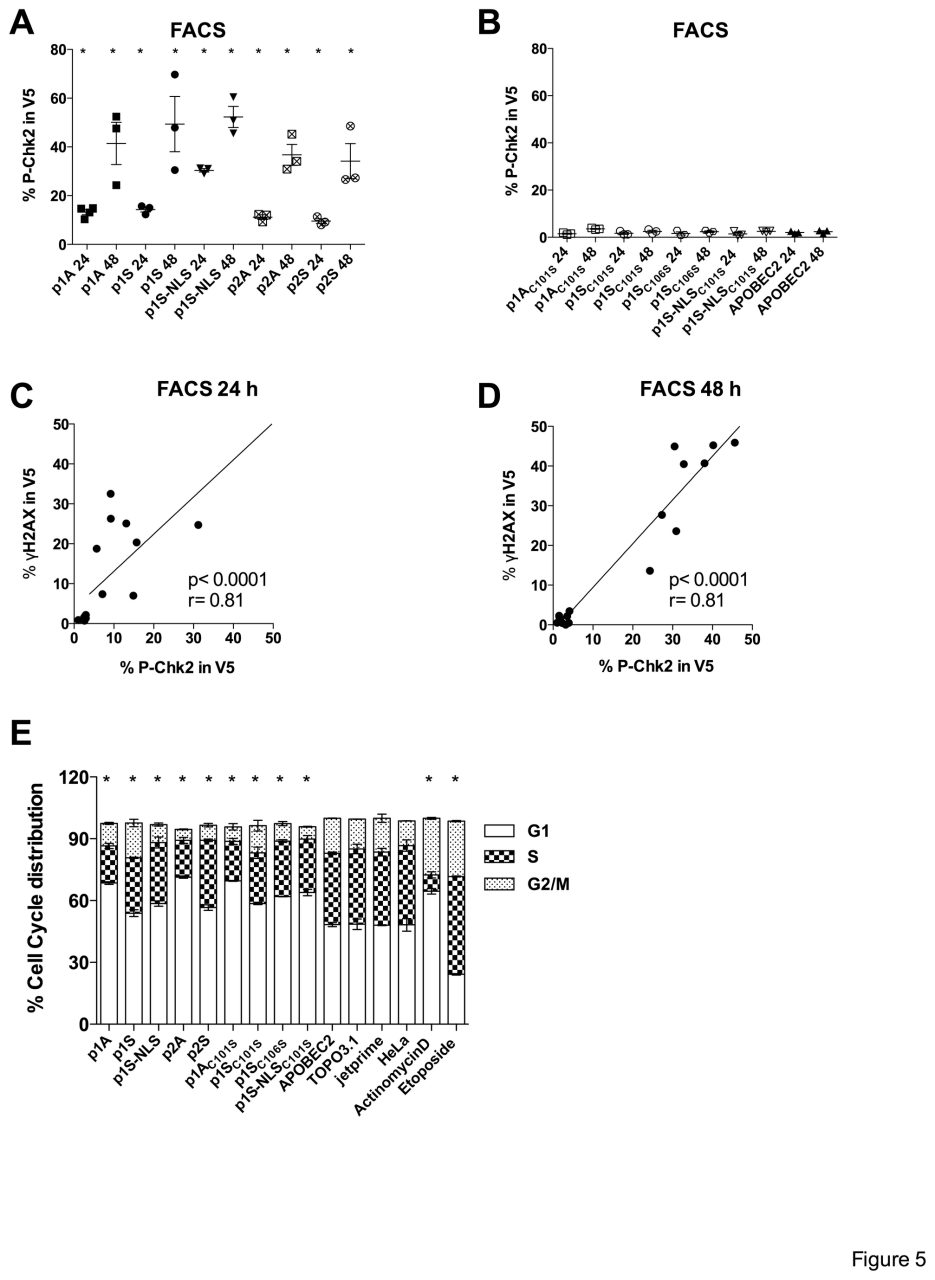
A3A expression induces DNA damage response and cell cycle arrest. (A) (B) Results illustrating percentage of P-Chk2 in V5 expressing cells at 24 and 48 h post-transfection. Mean and SEM are shown. Group comparisons to APOBEC2 at 24 and 48 h were calculated using the Mann-Whitney test (*p < 0.05). (C) (D) Linear relationship of ©H2AX and P-Chk2 at 24 and 48 h post-transfection respectively. r, Spearman’s correlation coefficient; line shows nonlinear regression; p, P value. (E) Twenty-four hours post-transfection RNA was removed with RNase A and DNA was stained with propidium iodide (PI) prior to analysis by flow cytometry. Graph shows percentage of cell cycle distribution from three independent experiments. Cellular aggregates and debris were excluded from analysis by proper gating. Data were fitted to define the G1, S, G2/M phases by using the Dean-Jett-Fox mathematical model of the FlowJo software. The data for 100 µM actinomycin D and etoposide (positive controls) were taken at 16 h. Mean and SEM are shown. Differences in G1 phases were compared to APOBEC2 and were calculated by using the Mann-Whitney test (*p < 0.05).

After DNA damage human cell cycle checkpoint kinase 2 (Chk2) is activated by phosphorylation of Thr68 mediated by ATM/ATR kinases [69–72]. Activated P-Chk2 inhibits CDC25C phosphatase, preventing entry into mitosis and leading to cell cycle arrest in G1 phase [73]. To investigate P-Chk2 involvement, HeLa cells were transfected with the A3A constructs and analysed by flow cytometry with 100 µM etoposide treated cells serving as positive control. P-CHK2 was detected for all functional constructs with highest levels found for p1S-NLS (Figure 5A). No P-Chk2 were observed in cells transfected with catalytic inactive mutants, APOBEC2 (Figure 5B) as well as TOPO3.1 vector and non-transfected cells. Indeed, the results are in remarkable agreement with the ©H2AX data (Figure 5C and D).

Since activation of Chk2 is associated with cycle arrest, we analysed the distribution of cell cycle phases in A3A transfected HeLa cells by propidium iodide (PI) staining and flow cytometry. At 24 h the distribution for non-transfected and transfection negative controls (TOPO3.1 or APOBEC2) was ~45-50% in G1, ~35-40% in S and ~12-17% in G2/M phase (Figure 5E). Interestingly following A3A transfection, a majority of cells were in G1 (56-70%), indicating cell cycle arrest at G1/S. The actinomycin D and etoposide positive controls are shown to the right (Figure 5E).

### A3A expression leading to cell death

**Figure 6:**
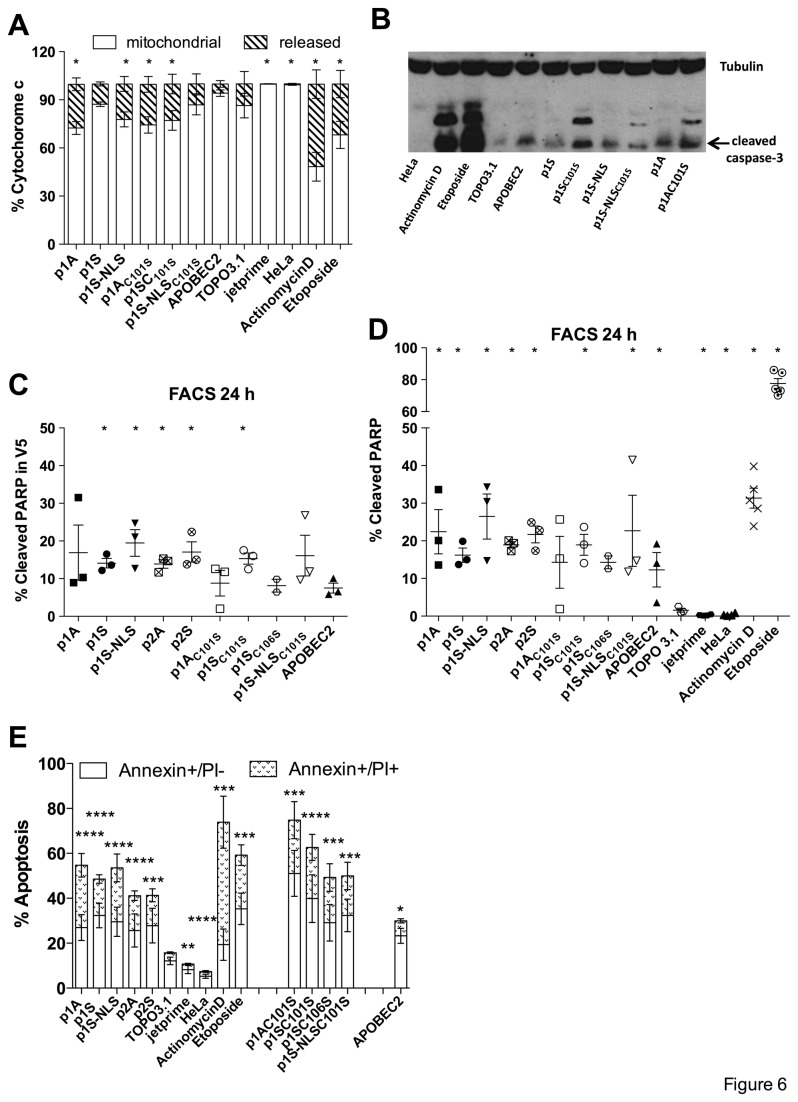
A3A over-expression triggers intrinsic apoptotic pathway. (A) FACS analysis of cytochrome c release (striped) in HeLa cells 24 h post-transfection. Treatment by 100 µM of actinomycin D and 100 µM etoposide served as positive controls and were measured at 16 h. Means and SEM are given for three independent transfections. Differences in mitochondrial cytochrome c content were compared to APOBEC2 and calculated by using the Mann-Whitney test (*p < 0.05). (B) Western blot analysis of cleaved caspase-3 levels at 24 h post transfection. Beta-tubulin was used as loading control. (C) FACS analysis of cleaved PARP in V5 expressing cells. Mean and SEM are shown for 2-3 independent experiments. Group comparisons to APOBEC2 were calculated using the Mann-Whitney test (*p < 0.05). (D) FACS analysis of cleaved PARP in total cells. Mean and SEM are shown for 2-5 independent experiments. Group comparisons to TOPO3.1 were calculated using the Mann-Whitney test (*p < 0.05). (E) FACS analysis of early apoptosis (Annexin V positive, PI negative cells - white) and late apoptosis/necrosis (Annexin V, PI double positive - patterned) 24 h post-transfection. Means and SEM are given from five independent experiments. Differences in early and late apoptosis were compared to TOPO3.1 and calculated by using the Mann-Whitney test (*p < 0.05; ***p< 0.001).

To assess whether apoptosis may follow A3A induced DNA damage, we analysed cytochrome c release, caspase-3 activation, PARP cleavage and phosphatidylserine exposure all markers of the intrinsic apoptotic pathway. Transfected HeLa cells were analysed by flow cytometry. Increased amounts of released mitochondrial cytochrome c were observed in cells transfected with A3A compared to APOBEC2 control (Figure 6A). However, the A3A catalytic mutants also induced cytochrome c release. To investigate whether cytochrome c release leads to caspase-3 activation, total protein was analysed by Western blotting and incubated with an antibody against cleaved caspase-3. Cleaved caspase-3 was found for all A3A constructs, however at levels comparable to the TOPO3.1 and APOBEC2 negative DNA controls (Figure 6B).

PARP is a 116 kDa nuclear polyADP-ribose polymerase involved in DNA repair following stress [74]. PARP can be cleaved by ICE-like caspases *in vitro* [75,76] and is one of the main cleavage targets of caspase-3 *in vivo* [77,78]. Intact PARP allows cells to maintain their viability and cleavage of PARP represents a marker for cellular apoptosis [79]. By FACS analysis using an antibody to cleaved PARP, we found cleaved PARP in varying degrees in cells transfected with several constructs compared to APOBEC2 control (Figure 6C). After applying the percentage of cleaved PARP from the entire cell population, even the APOBEC2 control showed significantly increased PARP levels compared to the empty vector TOPO3.1 (Figure 6D). Moreover, untransfected cells and cells treated only with the transfection agent jetprime showed less amounts of cleaved PARP compared to cells transfected with TOPO3.1, indicating an impact of transfected DNA on apoptosis induction (Figure 6D).

The redistribution of negatively charged PS to the outer leaflet of the cellular membrane represents another marker for the detection of early apoptosis [80,81]. Annexin V, a 36 kDa phospholipid binding protein recognizes PS on cell surfaces of early apoptotic cells [80]. We investigated the redistribution of PS in A3A transfected HeLa cells with Annexin V by flow cytometry. Dead cells were excluded by additional staining with PI. Figure 6E shows data in percentage of early apoptosis (Annexin positive and PI negative cells) and late apoptosis/necrosis (Annexin V and PI double-positive cells). Compared to TOPO3.1, all constructs scored positive for apoptosis including the cysteine mutants and APOBEC2 (Figure 6E). As seen for PARP, cells transfected with TOPO3.1 again showed increased apoptosis induction over untransfected cells and those treated only with the transfection agent jetprime (Figure 6E).

**Figure 7:**
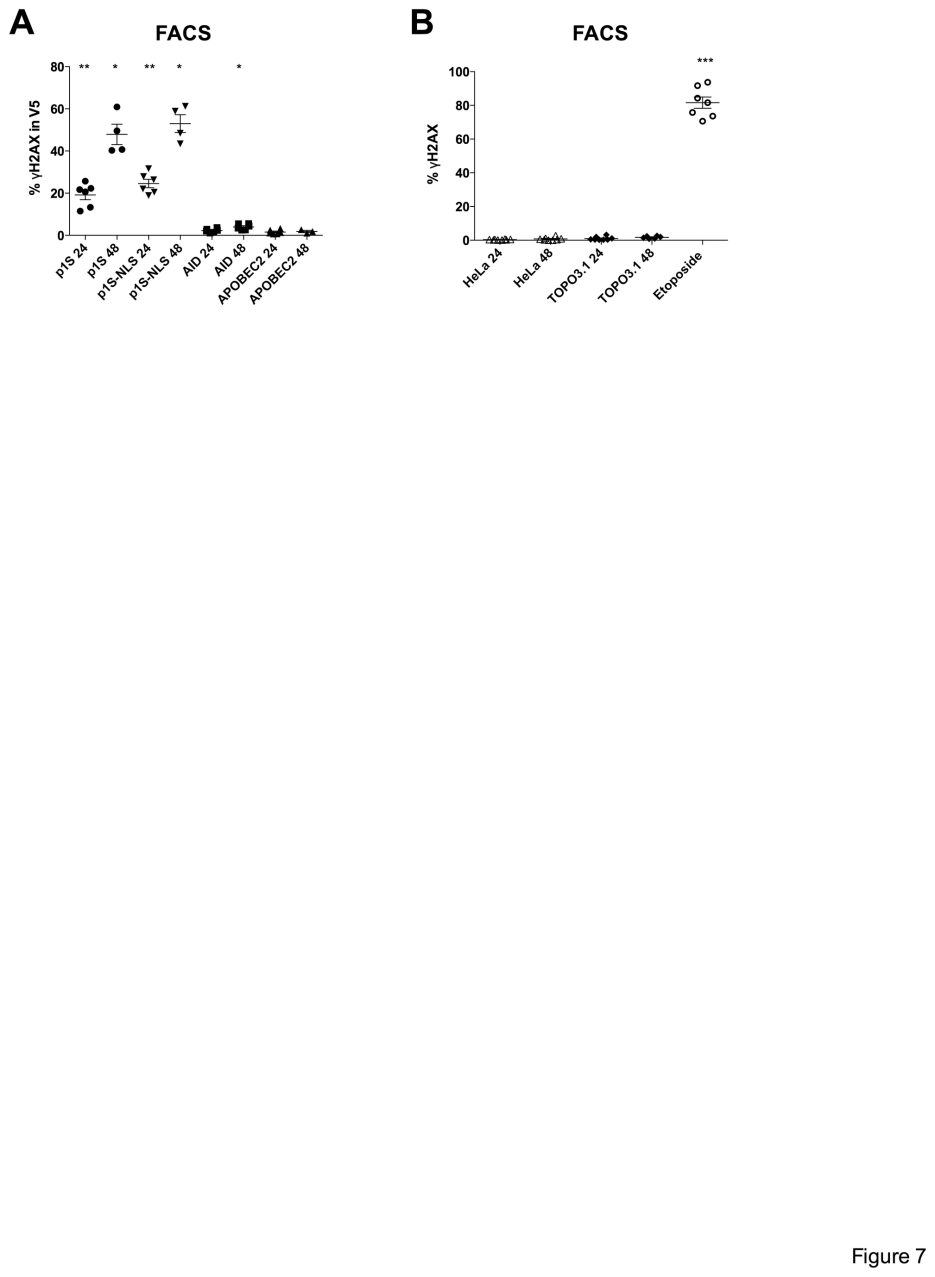
No induction of DSBs by AID expression. (A) Results illustrating percentage of γH2AX in V5 expressing cells at 24 and 48 h post transfection. Group comparisons and differences to APOBEC2 at 24 and 48 h were calculated using the Mann-Whitney test (*p < 0.05; **p< 0.01). (B) Graph illustrates percentage of ©H2AX in cells at 24 and 48 h for transfections with TOPO3.1 empty vector control. Incubation for 16 h with 100 µM with DSBs inducing drug etoposide served as positive control. Dots are representative for independent experiments. Mean and SEM are shown. Group comparisons were calculated using the Kruskal-Wallis test (***p< 0.001).

Given that targeted AID generated DSBs is the paradigm for human polynucleotide cytidine deaminases, it would be useful to situate AID in the present context. Accordingly, we analyzed over expression of a functionally active V5 tagged human AID construct cloned in the same vector [29,82,83]. At 24 and 48 h post-transfection of HeLa cells a few γH2AX positive cells were noted, but not more than for the APOBEC2 over expression control (Figure 7A). These results are in sharp contrast to the proportion of cells showing DSBs following transfection of p1S and p1S-NLS plasmids or treatment with etoposide (Figure 7A and B).

## Discussion

Our results demonstrate that both A3A isoforms can translocate to the nucleus and cause DNA damage – both cytidine hypermutation and DSBs. As the levels of γH2AX reflect the amount of DSBs both A3A isoforms seem to be equally efficient. The translocation levels for p1S-NLS are as high as p1S emphasizing the natural potential of A3A to transfer to the nucleus and perhaps to saturation. Not surprisingly A3A-induced DSBs are dependent on deaminase activity (Figures 2B and 3A) while UNG initiates base excision repair as cells co-transfected with A3A and the uracil-N-glycosidase inhibitor (UGI) showed lower levels of DSBs and parallels the findings for A3A hypermutation of nuDNA (Figure 3D) [40]. The râisons d’être for encoding two isoforms is not evident especially as the chimpanzee, bonobo and gorilla genomes encode only the p2 isoform with an adequate Kozak motif. Other monkey genomes show strong Kozak motifs surrounding the p1 or p2 initiator codons [84].

DSBs are considered to be biologically significant because their repair is more difficult compared to other types of DNA damage and DSBs are associated with a higher risk of mutagenicity or activation of apoptotic programs. The enormous amounts of A3A induced DSBs detected probably overwhelm DNA repair - up to 50% of DSBs were still not repaired by 48 hours – so leading to apoptosis [85]. This conclusion is reinforced by the observation that targeted AID induced breaks are invariably repaired by 24 hours. It may be argued that the above observation pertains to targeted AID in physiologically relevant system. However, AID over expression failed to yield detectable DSBs above controls (Figure 7A) indicating that AID and A3A are not equivalent. This contrast suggests that A3A accesses nuDNA in a non-targeted manner.

The degree of editing of *CMYC* or *TP53* DNA in interferon-〈 treated activated primary CD4+ T lymphocytes is comparable to that found for A3A transfected 293T-UGI cells (Figure 4) [40]. We make extensive use of 3DPCR, which selectively amplifies AT rich DNA – and A3A edited nuDNA [40,58]. Despite this we were unable to recover hypermutated DNA from PHA+IL2 activated CD4+ lymphocytes even though they showed comparable levels of DSBs. This apparent conundrum can be appreciated when it is realized that i) T cell contraction following a strong stimulus can generate DSBs [86], ii) IFN-〈 strongly induces *A3A* transcription while A3B is hardly affected [34,39,61] and iii) that 3DPCR generally recovers extensively hyperedited DNA, something of the order of >10% of cytidine targets which reduces to a few per hundred total bases, for example Figure 4E. In short it is not a quantitative technique. The observation for activated purified CD4+ T lymphocytes is very important for it is the first time we have detected A3A mediated hypermutation of nuDNA from primary cells of patients without known disease. That the DSBs are still detectable at 48 hours indicates that mismatch repair may well be overwhelmed and cannot repair them (Figure 4A and B).

Compared to normal cells, cancer cells generally display increased levels of γH2AX, hence more DSBs [87–92]. Some studies even suggest γH2AX quantification can be used for the detection of precancerous lesions [55,89,93]. While it would certainly be erroneous to ascribe all DSBs to A3A activity, it now becomes a variable especially in pathologies with an inflammatory component. Impaired DNA repair results in accumulated DNA damage [94] and has also been linked with aging [95–98]. Next to telomere erosion, induction of DSBs associate with increased γH2AX foci and impaired DDR are common events in mammalian aging [99–101]. More γH2AX were observed in cells undergoing accelerated aging taken from patients with Werner syndrome [102,103]. Accumulation of unrepaired DSBs is further linked with cellular senescence featured by irreversible cell cycle arrest, which on the one hand prevents tumour formation but on the other hand promotes aging [101,104,105].

The pro-apoptotic activity of the A3A catalytic mutants was intriguing and probably reflects non-physiological activity - the mutants very likely behave as ssDNA binding proteins, which can impact the cell cycle leading to cell stress and death. The induction of apoptosis has been described after enhanced DNA binding of Sp1 [106] or ruthenium (II) polypyridyl complex [107]. Further it is known that DNA binding of the bisbenzimide Hoechst 33342 inhibits the activity of transcription and replication [108] and induces apoptosis in several cell lines [109–111]. Accordingly, these A3A mutants are not null-mutants and must be used with care. Apart from this, transfected DNA itself as well as protein over-expression can trigger apoptosis as seen from cells transfected with empty TOPO3.1 vector and APOBEC2 (Figure 6).

The revolution in cancer genomics is showing far more mutations and rearrangements that hitherto expected. Apart from the singular cases involving UV or smoking related cancers, CG->TA appears to be the dominant mutation. In addition some genomes exhibit what is called chromothrypsis, or chromosome shattering, where phenomenal numbers of rearranged DNA segments are apparent. Chromothrypsis is also accompanied by somatic mutations [112]. More recently local hypermutation, or kataegis, has been described in breast cancer genomes. Again the dominant mutation is CG->TA [113]. The strong association of C->T transitions with the TpC dinucleotide suggests an APOBEC3 enzyme. While the relative contributions of A3A and A3B need to be worked out, cancer can emerge on an *A3B* ^*-/-*^ background [44,45]. Hence, this strong TpC bias, very reminiscent of A3A hyperediting, suggests that the dominant mutation in cancer genomes is actually the C->T transition, with G->A transitions simply reflecting this mutation on the other strand [40].

This finding suggests that, apart from the special cases cited above, the dominant cancer mutation could well be the C->T transition. However, cancer genomes reflect the ravages of mutation and DNA repair. Interestingly there is an even greater bias in favour of CG->NN somatic mutations in cancer genomes as opposed to TA->NN. It is possible that numerous CG->TA mutations could have been initiated by A3A deamination, yet their origins obscured by DNA repair. More recently efficient A3A editing of 5-methylcytidine has been described including two 5meCpG sites in the *TP53* exon 8 sequence [114–116]. Coupled with DSB breaks it is clear that one enzyme, A3A, is in principle capable of explaining the four hallmarks of cancer genomes -i) huge numbers of mutations, ii) most of which are CG->TA and CG->NN, iii) 5MeCpG hotspots and iv) double strand DNA breaks.

Clearly human A3A can be a danger to cellular genomic integrity. The *A3A* precursor has been traced back some 140 Myr and cuts across the primate, carnivore and artiodactyl orders [14] suggesting that the physiological role of the A3A enzyme has been well honed. To date no human *A3A* ^*-/-*^ genotype has been described. The contrast between AID and A3A is striking, the more so as the *A3* locus presumably arose by duplication of the older *AID* gene. AID enzyme has lower catalytic activity [117] and is targeted to discrete loci in B cells, the DNA breaks made being rapidly repaired with off target deamination is something of a rarity. It appears that A3A has undergone selection for extremely efficient non-targeted cytidine deamination of nuDNA. Local A3A induced mutation rates that can reach something between 0.1 and 0.8 per base [40], which is more than enough to push the cell beyond the error threshold, a well known concept to RNA virologists [118–120], into some form of caspase-3 independent cell death. Recently, we described TRIB3 as a pseudokinase that degrades A3A so protecting the genome [121]. Intriguingly it is part of the CtIP-Rb-BRCA1-ATM protein network. It is remarkable that such opposites, genome stability and A3A hypermutagenesis, are part of the same network, as though the latter might possibly be a mechanism of last resort in the case of genetic conflicts. The hypermutated nuDNA and DSBs identified in INF-α treated CD4+ T lymphocytes suggests that A3A induced DNA damage is far from a rare phenomenon. The pathological consequences of dysfunctional *A3A* gene control need to be explored especially as many cancers emerge in a background of chronic inflammation.
